# Systemic treatment for primary malignant sarcomas arising in craniofacial bones

**DOI:** 10.3389/fonc.2022.966073

**Published:** 2022-09-08

**Authors:** Stefan S. Bielack

**Affiliations:** ^1^ Pediatrics 5 (Oncology, Hematology, Immunology), Center for Pediatric, Adolescent and Women’s Medicine, Stuttgart Cancer Center, Klinikum Stuttgart–Olgahospital, Stuttgart, Germany; ^2^ Department of Pediatric Hematology and Oncology, University Children’s Hospital Muenster, Muenster, Germany

**Keywords:** osteosarcoma, Ewing sarcoma, chondrosacoma, craniofacial, chemotherapy, cancer

## Abstract

**Introduction:**

Craniofacial bones may be the site of origin of various sarcomas. We review the various malignancies affecting this region of the body and attempt to put systemic treatment approaches into perspective.

**Material and methods:**

Non-systematic literature review

**Results:**

Conventional types of osteosarcoma, Ewing sarcoma, and chondrosarcoma are the most frequent bone sarcomas occurring in craniofacial region, but variants may occur. The tumors’ biologies and the resulting treatment strategies vary distinctly. As a general rule, local control remains paramount regardless of histology. The efficacy of antineoplastic chemotherapy varies by type of malignancy. It is clearly indicated in Ewing sarcoma and related tumors, potentially of benefit in high-grade osteosarcoma, undifferentiated pleomorphic sarcoma, dedifferentiated and mesenchymal chondrosarcoma, and of no proven benefit in the others.

**Conclusions:**

Various histologies demand various and distinct treatment approaches, with local control remaining paramount in all. The efficacy of systemic treatments varies by type of tumor. Prospective trials would help in all of these to better define systemic treatment strategies.

## Introduction

Systemic therapy is an integral part of therapy for some of the most common malignant bone tumors. Successful approaches were, however, generally developed outside of the head and neck. They may not always apply to this region of the body without modifications. This review aims to focus on systemic therapies for the various histotypes of craniofacial bone sarcomas, especially where it deviates from other sites of the body.

## Osteosarcoma

Osteosarcoma is the most frequent primary bone cancer. It mainly affects teenagers and young adults and there the extremity bones (1, 2). Most osteosarcomas are high-grade, but there are some rare low-grade malignancies which carry a lower risk of systemic spread and are treated by surgery alone. The further text refers to high-grade tumors. These carry a very high risk of metastases, mainly to the lungs, rarer to distant bones or even other sites (1, 2). Micrometastases are likely even if the disease appears localized at diagnosis.

Accordingly, local therapy of the primary alone will rarely lead to cure. Only the introduction of chemotherapy into a multimodal treatment context resulted in frequent cure (3). Doxorubicin, high-dose methotrexate, cisplatin, and ifosfamide are considered the most effective agents. Local treatment, however, remains essential. Surgery must result in wide margins. The whole tumor, covered by an unviolated cuff of soft tissue, must be removed in one piece (4). With surgery and intensive chemotherapy, 60-70% of patients with seemingly localized extremity disease may be cured (5). The outlook is much worse for patients with primary metastases (6).

With below 5-10% of all primaries, the craniofacial bones rank among osteosarcoma´s rarer sites. The median age of affected patients is far older than in the more common extremity locations (7, 8). There is a preponderance of secondary malignancies at this location, particularly after prior radiotherapy (9). Radiotherapy was then often administered for other cancers. The most notable example is radiotherapy given for retinoblastoma (10, 11). Other cancers, however, may also have been present.

Local treatment principles appropriate for extremity osteosarcoma also apply to the head and neck region. There, local control poses much more of a challenge than in the limbs, as amputation cannot be an option even for the most extensive lesions. Owing to the reduced ability to achieve wide surgical margins (4), the local failure rate is much higher than usual. Radiotherapy can only be a substitute for surgery if administered at very high dosages, probably exceeding 60-70 Gy. Such doses are more likely to be reached with proton or heavy ion irradiation (12–16), which may offer an option for selected craniofacial osteosarcomas.

Craniofacial osteosarcomas may not metastasize quite as frequently as their extremity counterparts. This may be due to more of the tumors having a low-grade histology. Hence, the use of systemic chemotherapy is not universally accepted (17). A true multitude of usually small mono-institutional series has described outcomes with varying forms of treatment. In such analyses, none, some, or all craniofacial osteosarcomas may have been treated with chemotherapy, other therapies may have differed. It is nay impossible to make any clear deductions about the role of antineoplastic therapy from these reports.

At the end of the last millennium, a meta-analysis of the literature finally pointed to a potential beneficial effect of adjuvant treatment, although its magnitude remained a matter of debate (18). Another meta-analysis, published almost simultaneously, came to quite the opposite conclusion (19). A summary of these and other relevant publications investigating the matter is presented in **Table 1**. The reported outcomes are puzzling and any opinion will find a report to support it. This may be so because investigators may have been inclined to cure local therapeutic inadequacies by systemic chemotherapy. This, however, is bound to fail at any site.

**Table 1:** Five-year survival of patients with craniofacial osteosarcoma receiving or not receiving chemotherapy.

Reference	site	chemotherapy	survival	comments
**Larger single institution experiences**				
Patel 2002 (20)	craniofacial	30 CT	77%^a^ ^,^ ^b^	histologic response unfavorable in 22/30 chemotherapeutically treated patients
		14 no CT	91%^a^ ^,^ ^b^	negative surgical margins only significant predictor of survival
Guadagnolo 2009 (21)	craniofacial	63 CT	57%	local failure rate greater threat than systemic spread, use of chemotherapy more
		56 no CT	70%	likely with the most adverse presentations (negative selection bias)
Chen 2017 (22)	craniofacial	38 CT	47%	adjuvant chemotherapy potentially improved survival when regarding prognostic factors
		119 no CT	52%	
**Multi-institutional series**				
Canadian Society 2004 (23 *)*	gnathic	17 CT	ca. 70%^c^	trend favoring chemotherapy, local recurrence more common than metastases
		15 no CT	ca. 55%^c^	positive margins most strongly associated with prognosis
Jasnau 2008 (24 *)*	craniofacial	44 CT	75%	extra-gnathic site and postsurgical tumor rests unfavorable
		5 no CT	67%	
Thariat 2012 (25 *)*	mandible	91 CT	69%^d^	disease-related survival better without neoadjuvant chemotherapy
		20 no CT		
Bouaoud 2019 (26 *)*	craniofaciall	21 CT	79%^a^	patients treated with neoadjuvant chemotherapy, no statistical difference
		10 no CT	67%^a^	
Smith 2003 (27 *)*	craniofacial	129 CT	71%	only operated patients, only patients not receiving radiotherapy
		153 no CT	75%	
Boon 2017 (28 *)*	craniofacial	13 CT, CS	ca. 75%^c^	chemotherapy increased local relapse-free interval upon multivariate testing
		10 no CT, CS	ca. 75%^c^	
		16 CT, IS	ca. 50%^c^	
		11 no CT, IS	ca. 25%^c^	
Shim 2021 (29 *)*	craniofacial	70 preop. CT	ca. 62%^c^	trend favoring combined pre- and postoperative chemotherapy
		38 pre- & postop. CT	ca. 65%^c^	
		122 CT, RT	ca. 55%^c^	
		305 no CT/RT	ca. 58%^c^	
Merna 2021 (30 *)*	skull-base	114 CT	ca. 65%^c^	chemotherapy without significant effect upon uni- and multivariate testing
		82 no CT	ca. 65%^c^	
Smeele 1997 (18 *)*	craniofacial	78 no CT, complete S	ca. 60%^c^	overall- and event-free survival improved with chemotherapy
		27 CT, complete S	ca. 80%^c^	
		42 no CT, incomplete S	ca. 15%^c^	
		33 CT, incomplete S	ca. 40%^c^	
Kassir 1997 (19 *)*	craniofacial	71 no CT	46%	surgery alone better than when combined with chemotherapy
		23 no CT, RT	20%	
		12 CT	50%	
		13 CT, RT	67% (15 mo.)	

Redundancy between publications possible.

a event-free survival.

b at 3 years.

c estimated from figure.

d all patients, distinction between chemotherapy and none not reported.

CT, chemotherapy; CS, complete surgery; IS, incomplete surgery; pre- & postop, preoperative and postoperative; RT, radiotherapy; S, surgery.

With the data available, the general view has become that patients affected by craniofacial osteosarcomas benefit to some extent from systemic chemotherapy. Accordingly, the current European guidelines favor a multimodal - local plus systemic - approach (5). The choice of drugs then resembles that used against its extremity counterpart (2). It must be noted that older adults do often not tolerate high-dose methotrexate, an integral part of chemotherapy in the young (31). Ifosfamide may offer a reasonable substitute (32). Patients over the age of 65 generally tolerate chemotherapy very poorly and there is no evidence-base on which to decide if and which regimen to use. Single agent doxorubicin may be one option, as may be others.

The response of an osteosarcoma to preoperative chemotherapy has been shown to be a major prognostic factor (33). Predicting this response can be of value in helping to decide the next therapeutic steps. Various methods exist, none is close to perfect. An analysis specifically focusing on head and neck osteosarcomas was able to demonstrate that ^18^FDG PET/CT was more reliable than standard imaging in evaluating response to neo-adjuvant chemotherapy in this location (34).

The administration of targeted therapies, especially tyrosine kinase inhibitors, may prolong life for a few months in patients with unresectable extremity osteosarcomas (35–40). Such therapy may also be considered for craniofacial lesions. Unfortunately, targeted therapies alone will never be curative in any location.

## Undifferentiated pleomorphic sarcoma (UPS)

Sometimes, tumors which would otherwise be classified as osteosarcoma do not produce any osteoid. These lesions are then characterized as undifferentiated pleomorphic sarcoma (UPS; formerly: malignant fibrous histiocytoma, MFH) (41). Their overall treatment strategy, prognostic factors and outcomes closely resemble that of its more frequent counterpart, osteosarcoma, even though the tumor response rate to chemotherapy seems to be lower (42, 43). Without relevant data about craniofacial primaries, it seems appropriate to treat such tumors just as one would osteosarcomas, with systemic chemotherapy.

## Ewing sarcoma

Ewing sarcomas are fully malignant tumors which may arise in bone or, rarer, soft tissues. Primary sites can be in the extremities or axial skeleton. Tumors mostly affect young adolescents, but may occur at any age (44). Treatment can only be successful if a combination of local (surgery, radiation, or both) and systemic chemotherapy is administered. Current regimens include an anthracycline, generally doxorubicin, alkykators, vincristine, and etoposide (45). The so called augmented interval-compressed VDC/IE-scheme (vincristine, doxorubicin, cyclophosphamide/ifosfamide, etoposide) (46) may be more efficacious and less toxic than the VIDE-regimen (vincristine, ifosfamide, doxorubicin, etopside), which was previously used in many European counties (47). High-dose chemotherapy with stem-cell rescue has its role in some highly selected patients (47). If treated appropriately, some two thirds of affected individuals may become long-term survivors (44).

Ewing tumors primarily affecting craniofacial bones are detected in no more than five percent of patients (48). In the American Intergroup Ewing’s Sarcoma Study (IESS), for instance, it was reported that this osseous site was affected in approximately 4% of primaries (49). Craniofacial Ewing sarcomas, hence, may be considered rare even in the largest treatment centers. As an example, the University of Florida reported only eight such individuals observed over a 40-year period (50).

Ewing sarcomas of the craniofacial bones have been the focus of multiple publications. Mono-centric reports include, for example, a series of 14 patients who were subjected to tumor surgery, adjuvant chemotherapy, and radiotherapy. Eight patients survived five years or longer (51). The University of Florida reported no more than nine chemotherapeutically treated patients, of which one was clearly of extraosseous origin. Two thirds became 10-year survivors (50). A study from Toronto focused on the sino-nasal tract and maxillary bone and reported eight affected patients with follow-up data, of whom six survived for 1 – 158 months, 5 without evidence of disease (52). An Indian group reported an event-free survival of 59% at five years for 25 chemotherapeutically treated osseous primaries of the head and neck, three of which had metastases (53). The Mayo Clinic published 17 chemotherapeutically treated craniofacial Ewing sarcomas, five of these in the cervical spine, two with primary metastases. Five-year overall survival was given as 87% (54).

A summary of multi-centric analyses is presented in **Table 2**. The Intergroup Ewing’s Sarcoma Study (IESS) reported that head and neck tumors comprised only 4% of all primaries, the gnathic bones being most commonly affected. Their prognosis was found to be significantly better than that of Ewing sarcoma in general (49). The Italian Association of Pediatric Hematology and Oncology reported a ten year actuarial survival of 64% in 21 multi-modally treated pediatric patients with localized Ewing sarcomas of the craniofacial bones (55). The German-Dutch Gesellschaft für Pädiatrische Onkologie und Hämatologie (GPOH) reported 51 craniofacial Ewing sarcomas treated on the E.W.I.N.G.-99 trial. Their median age was 12 years. Approximately nine out of ten tumors were of osseous origin. Three-year event-free survival was 74% for 44 patients with localized disease (56). Forty-seven French patients were registered on the same Euro-E.W.I.N.G.-99 trial, 42 of those arose within bone. Primary metastases were observed in less than 10% of affected individuals. Three-year, event-free survival was reported as 79% (57).

**Table 2:** Overview of multi-institutional analyses of chemotherapy in Ewing sarcoma of the head and neck.

Reference	Site	pts	survival	comments
Siegal 1987 (49)	craniofacial	29	ca. 70%^a^	pooled data from 4 Intergroup Ewing’s Sarcoma studies
Berger 2013 (55)	craniofacial	21	64%^b^	7/17 evaluable patients with good tumor response
Grevener 2016 (56)	craniofacial	44	ca. 70%^a^	12/15 evaluable patients with good tumor response
Bouaoud 2016 (57)	craniofacial	43	91%^c^	many late sequelae
Thorn 2016 (58)	maxilla incl. sinus	79	86%^d^	literature review, isolated patients treated without chemotherapy possible
Ellis 2017 (59)	craniofacial	183	54%^b^	SEER analysis, including patients with soft-tissue primaries and primary metastases
Martin 2019 (60)	skull	80	69%	SEER analysis, including 2 metastatic cases
Martin 2019 (8)	craniofacial	127	73%^e^	SEER analysis, pediatric patients only, 75% osseous primaries
Torabi 2020 (61)	craniofacial	187	75%	NCDB-database, all patients received chemotherapy
Rehman 2022 (48)	craniofacial	70	55/70	literature review of 71 studies, osseous primaries, only chemotherapeutically treated patients

Redundancy between publications possible.

a estimated from figure.

b at 10 years.

c at 3 years.

d observation period not specified.

e 8% treated without chemotherapy.

pts, patients.

As for reports about pooled data, Thorn et al. reviewed the published literature on Ewing tumors of the maxilla and maxillary sinus and found 93 cases. Over 90% of patients received combined local and systemic treatment, 70/79 individuals with any follow-up information remained alive, 68 of these disease-free (58). A Surveillance, Epidemiology, and End Results Program (SEER)-analysis of 183 craniofacial primaries found a lower tumor size and metastatic rate and a superior survival rate for craniofacial compared to other primaries (59). Another SEER-analysis identified 127 pediatric patients with Ewing sarcomas of the head and neck, of which some three quarters were of osseous origin. Five-year overall survival was reported as 72.9%, without any effect of age, sex, or irradiation (60). Focusing on 80 Ewing sarcomas of the skull, the same authors observed a five-year survival of 69% (8). A review of the National Cancer Database (NCDB) found 75% of chemotherapeutically treated patients with craniofacial Ewing sarcomas to survive beyond five years (61). Finally, a review of pertinent publications showed 55 of 70 chemotherapeutically treated individuals with craniofacial tumors to survive (48).

Recently, a rare malignant tumor characterized by the EWSR1:Friend leukemia integration 1 (FLI1)-translocation and complex epithelial differentiation was described and characterized as adamantinoma-like Ewing sarcoma (62). In even more uncommon instances, it seems to arise from osseous sites. Awareness of the morphologic and immunohistochemistry spectrum of this tumor is definitely required, but there is no justification for treating it differently from conventional Ewing sarcoma. It remains to be determined whether these tumors are only morphologically or also clinically distinct.

In summary, craniofacial location *per se* does not provide any reason to modify systemic Ewing sarcoma treatment, that is: All patients are to receive (neo-)adjuvant chemotherapy. If treated such, the prognosis of patients with craniofacial Ewing sarcomas may even be somewhat better than in other sites. It must be noted that, due to a limited amount of soft tissue able to hide tumor growth, craniofacial Ewing sarcomas are usually rather small when detected. As tumor size is an important prognostic factor in this disease (44, 45), this probably leads to a prognostic benefit for craniofacial primaries.

## Ewing-like sarcomas

In recent years, several molecularly defined tumors which morphologically resemble Ewing sarcoma but carry distinct genetic translocations have been identified (63–65). Among these, protein capicua homolog (CIC)- and B-cell lymphoma 6 corepressor (BCOR)-rearranged sarcomas along with sarcomas carrying Ewing sarcoma breakpoint region 1 (EWSR1)-non-erythroblast transformation specific (ETS) fusions feature most prominently. A growing variety of others is also being described.

The tumor biology and the clinical course associated with these heterogeneous malignancies vary greatly. Tumors with CIC-rearrangements usually arise in adults and seem to behave even more aggressively than classical Ewing tumors. They are hence associated with a particularly poor outcome (66). Primaries often involve the soft tissues rather than bone and seem to affect craniofacial sites only very infrequently. The exact role of systemic therapy and the optimal regimen to be used are still open, even with tumors of the more frequent locations.

Tumors with BCOR-alterations, on the other hand, seem to have a more favorable outlook. Patients tend to be younger than those with CIC-fusions, with a male predominance (67). Many patients receive chemotherapy as for Ewing sarcoma. Again, specific information about craniofacial tumors is very hard to come by.

Even less can be said about other translocations. At present, all should be included in appropriate trials and registries to learn more about their respective behavior. Until that is achieved, there seems to be no reason to choose one particular systemic therapy regimen over another.

## Conventional chondrosarcoma

Conventional chondrosarcomas are mesenchymal cartilaginous tumors of, generally, older adults. A significant proportion have their origin not in bone, but in soft tissues. While many are not highly malignant initially, recurrences tend to be of a higher grade, pointing to the need for meticulous local therapy (5).

This tumor-type is considered largely resistant to systemic therapies. Treatment is therefore generally by surgery only, with radiotherapy and, particularly, cytotoxic chemotherapy reserved for truly desperate situations (5). Drugs employed then often resemble those used against osteosarcoma. A very recent review suggested that chondrosarcoma patients might benefit from antiangiogenic therapy and that tumors harboring isocitrate dehydrogenase 1 (IDH1)-mutations might benefit from treatment with IDH1-inhibitors. Mammalian target of rapamycin (mTOR)-inhibitors and tyrosine kinase inhibitors were suggested for relapsed disease (68).

Rarely, chondrosarcomas may also arise in craniofacial sites. It is challenging o delineate any therapeutic differences in relation to this location. A review of the literature on skull-base chondrosarcomas concluded that maximal safe resection followed by radiotherapy was the treatment of choice for Grade II and III lesions, while there was no current role for chemotherapy (69). A literature review of 161 sino-nasal chondrosarcomas suggested aggressive surgical resection as the most common treatment modality for this condition, with adjuvant radiotherapy being used for prevention of local recurrence after subtotal or total resection, but, again, did not elaborate on chemotherapy (70).

Larygeal primaries may form a distinct subgroup among head and neck chondrosacomas. They involve the cricoid, thyroid cartilage, epiglottis, or arytenoid cartilages and represent these structures’ most frequent malignancy. Treatment is again local, systemic chemotherapy being very rarely employed. Disease-specific survival at 10 years has been reported as 82% in a recent systematic review of 592 patients. Here, only.2% of patients received chemotherapy (71).

In summary, the role of chemotherapy for conventional craniofacial chondrosarcoma, even if high-grade, is close to zero.

## Dedifferentiated chondrosarcoma

Dedifferentiated chondrosarcoma is morphologically and clinically very distinct from its conventional counterpart. This malignancy arises from a conventional chondrosarcoma, which is often low-grade, by dedifferentiation. It may then resemble any type of spindle cell sarcoma. The tumor extremely often metastasizes and the prognosis is hence dismal (72).

The only collaborative, prospective trial of 57 eligible patients, 34 of those with primarily localized disease, recently suggested that a multi-drug chemotherapy regimen originally developed against osteosarcoma might have some efficacy in this condition. Median overall survival at five years was reported as 39%. However, the number of craniofacial primaries, if any, was not specified (73). Systemic treatment options may include immunotherapy in IDH1-mutant tumors. This is also still under discussion (68).

Data on systemic therapy for craniofacially located dedifferentiated chondrosarcomas is sparse to almost non-existent. It was suggested that chemotherapy improved survival in a series of only 6 such lesions of the skull-base (74), but the numbers were so small that it was difficult to draw any firm conclusions. However, there is also no evidence suggesting that these tumors would behave differently than in the rest of the body. So, if the evidence was considered sufficient, it should also be followed for craniofacial primaries.

## Mesenchymal chondrosarcoma

Mesenchymal chondrosarcoma is a malignant tumor which may arise intra- or extraosseously. It is a small cell malignancy containing well-differentiated cartilage. Of note, it seems to be a fusion driven tumor, with Hes Related Family BHLH Transcription Factor With YRPW Motif 1: nuclear receptor coactivator 2 (HEY1-NCOA2) and interferon regulatory factor 2 binding protein 2: caudal type homeobox 1 (IRF2BP2-CDX1) fusions having recently been described. The disease may cause very late recurrences which manifest well over a decade after initial presentation. Surgery remains the mainstay of treatment (75).

Previously, from a limited experience, it was believed that chemotherapy was not effective (76). This may have to be questioned. In an intergroup series of 15 chemotherapeutically treated pediatric patients, 6/15 tumors were of craniofacial origin, only 4 of all tumors were located intraosseously. Actuarial 10-year overall and event-free survival were 67% and 53%, respectively (77). A European multicenter analysis of patients aged 11-80 years found 13% of 113 cases to arise in the head and neck, of whom 53/96 with localized disease received chemotherapy. The median progression free and overall survival for all 96 were 7 and 20 years, respectively. Primary site did not affect survival. Here, chemotherapy administration was definitely associated with reduced risks of recurrence and death (78).

The available data now argues for adjuvant chemotherapy in this type of tumor, with little reliable data on craniofacial lesions in particular. The optimal drug combination to be employed has not been defined. Mostly, patients will be offered Ewing based or, rarer, osteosarcoma based approaches.

## Clear cell chondrosarcoma

Clear cell chondrosarcoma is another uncommon, slowly growing variant of chondrosarcoma which usually affects extracranial sites. There, the tumor is treated by local therapy only (79). There is no reason to do so differently for craniofacial primaries.

## Ultra-rare malignant bone tumors

In addition to those tumors discussed above, a variety of ultra-rare semi-malignant and malignant sarcomas may affect craniofacial bones. These are not further discussed here.

## Conclusions

The role of chemotherapy for craniofacial sarcomas of bone differs by histology. It is clearly indicated in Ewing sarcoma and its variants. It may be useful in high-grade osteosarcoma, undifferentiated pleomorphic sarcoma (UPS), dedifferentiated and mesenchymal chondrosarcoma. It is as of completely unproven value in conventional, myxoid, and clear cell chondrosarcoma.

## Author contributions

The author confirms being the sole contributor of this work and has approved it for publication.

## Funding

This study was supported by Förderkreis krebskranke Kinder Stuttgart e. V.

## Conflict of interest

SB reports having received personal funds from Ipsen, Hoffmann-La Roche, Bayer Healthcare, Boehringer-Ingelheim, EISAI, MAP Biopharma, all outside the submitted work.

## Publisher’s note

All claims expressed in this article are solely those of the authors and do not necessarily represent those of their affiliated organizations, or those of the publisher, the editors and the reviewers. Any product that may be evaluated in this article, or claim that may be made by its manufacturer, is not guaranteed or endorsed by the publisher.
